# Knowledge and Attitudes Toward E-cigarette Use: A Cross-Sectional Study From Saudi Arabia

**DOI:** 10.7759/cureus.49583

**Published:** 2023-11-28

**Authors:** Shalan Alaamri, Abdallah Y Naser

**Affiliations:** 1 Department of Medicine, College of Medicine, University of Jeddah, Jeddah, SAU; 2 Department of Applied Pharmaceutical Sciences and Clinical Pharmacy, Isra University, Faculty of Pharmacoepidemiology, Amman, JOR

**Keywords:** awareness, saudi arabia, knowledge, e-cigarette, attitude

## Abstract

Background

In light of the fact that electronic cigarette (e-cigarette) use is a newly emerging behavior in the Middle Eastern region, where tobacco consumption is widespread, it is imperative to examine the present state of e-cigarette awareness and attitudes toward e-cigarettes. The aim of this research is to investigate the level of knowledge and attitudes pertaining to the utilization of e-cigarettes within the context of Saudi Arabia.

Methodology

A cross-sectional online survey study was conducted between May and September 2023 to assess public knowledge and attitudes toward e-cigarettes in Saudi Arabia. This study utilized a previously validated questionnaire to assess the knowledge and attitudes of participants regarding e-cigarettes. Binary logistic regression analysis was used to identify predictors of being knowledgeable of and having a positive attitude toward e-cigarettes.

Results

A total of 422 participants were involved in this study. Around one-third of the study participants were current smokers (37.2%). The median knowledge score for the study participants was 13.00 (11.00-14.00), which reflects a high level of e-cigarette knowledge. The median knowledge score for the study participants ranged between 3.00 and 15.00. The median attitude score for the study participants was 3.00 (1.00-5.00), which reflects a negative attitude toward the use of e-cigarettes. The median attitude score for the study participants ranged between 0.00 and 12.00. Participants with a monthly income of 5,001 SAR and above were more likely to be knowledgeable about e-cigarette use (p < 0.05). However, binary logistic regression analysis did not identify any statistically significant predictor of positive attitude toward the use of e-cigarettes (p > 0.05).

Conclusions

The study population exhibited a significant level of knowledge regarding e-cigarettes, which was coupled with a prevailing negative attitude toward their use. The income level of individuals emerged as a significant predictor of e-cigarette knowledge. To obtain a comprehensive knowledge of the factors that contribute to negative attitudes toward e-cigarette usage, particularly among specific demographic groups, it is imperative to employ a qualitative research methodology.

## Introduction

Electronic cigarettes or e-cigarettes are vaporization devices powered by lithium-ion batteries and marketed as a novel smoking cessation aid. The primary benefits of vaping include (1) smoking cessation, (2) reducing cigarette consumption, (3) reducing tobacco craving, (4) mitigating harm when used as a substitute for conventional cigarettes, (5) being cheaper than conventional cigarettes, and (6) having a superior taste and odor compared to conventional cigarettes [1]. Although some e-cigarette brands do not contain nicotine [2], the vast majority deliver nicotine-containing vapor (4-20 mg/puff) along with flavorings such as propylene glycol and glycerin [3]. There is no reliable agreement as to whether e-cigarettes are an effective smoking cessation tool that is safer and healthier than its tobacco equivalents with a comparably lower or non-existent potential for dependence and/or addiction [4]. Diverse studies evaluating the efficacy of e-cigarettes as smoking cessation aides have produced contradictory results. A systematic review revealed that smokers using e-cigarettes as a smoking cessation tool were less likely to cease [5]. Another study found a significant increase in the use of e-cigarettes among young individuals who have never smoked [3]. Other studies, however, have demonstrated its efficacy as a smoking cessation tool. Tobacco consumers who were encouraged to transition to e-cigarettes smoked fewer cigarettes per day, became less dependent on cigarettes, and were more motivated to cease [6]. E-cigarettes were found to be less detrimental than cigarettes because they do not burn, do not contain tobacco, and their vapor contains fewer toxic compounds than tobacco [7]. It has also been demonstrated that e-cigarettes were intended as a replacement for tobacco cigarettes by the majority of smokers [8], who believed that e-cigarettes posed a lower risk of developing lung or oral malignancies or cardiovascular disease than tobacco cigarettes [9]. On the other hand, other studies have uncovered risk factors for the chronic use of e-cigarettes, casting doubt on the safety of this smoking device. However, the risk of developing cardiovascular and pulmonary conditions that are not malignant over the long term is comparable to that of smoking cigarettes. Moreover, identical to tobacco cigarettes, e-cigarette emissions were associated with both second-hand and third-hand smoking [10,11]. The safety of the liquid in e-cigarettes, e-liquid, is also unknown.

Given that e-cigarettes are comparatively new devices, assessing the knowledge and attitudes of individuals regarding their risks and benefits has become a topic of research interest in particular regions of the globe. Among adult populations in the United States, Puerto Rico, and elsewhere, two previous studies on e-cigarette knowledge and attitudes have been conducted [12,13]. Both investigations revealed a lack of knowledge about e-cigarettes, particularly regarding their regulations and constituents [12,13]. In the Middle East, where cultures and demographics are distinct from those of the West, the topic of e-cigarettes is still understudied. Given that e-cigarette use is a newly emerging habit in the Middle East, where tobacco use is prevalent, it is crucial to investigate the current level of e-cigarette knowledge and attitude toward e-cigarettes. The aim of this study is to examine the knowledge and attitudes toward e-cigarette use in Saudi Arabia.

## Materials and methods

Study design and settings

A cross-sectional online survey study was conducted between May and September 2023 to assess public knowledge and attitudes toward e-cigarettes in Saudi Arabia.

Sampling procedure

The study sample was collected using a technique of convenience sampling. This method of sampling is a form of non-probability sampling. This study included eligible participants who met our inclusion criteria and were willing to participate because of their availability. On the first page of the questionnaire, a consent form was included, and participants were informed that they may proceed or exit at that time. To make participants aware of the significance of their participation, the study’s objectives were written explicitly. The inclusion criteria were outlined in the study’s invitation letter.

Population studied

All individuals currently residing in Saudi Arabia were invited to participate in this study. The URL to the questionnaire was shared via social media websites (Facebook, Snapchat, and Instagram). Individuals who presently reside in Saudi Arabia and were at least 18 years old were eligible. There were no gender, occupation, or nationality restrictions.

Study tool

This study utilized a previously developed questionnaire to assess the knowledge and attitudes of participants regarding e-cigarettes [14]. The attitude section consisted of 13 yes or no questions that were devised based on an exhaustive literature review [1,8,9]. The attitude section examined participants’ attitudes toward e-cigarette use in places prohibiting other forms of smoking, its social acceptability, its effectiveness and acceptability as a smoking cessation tool, its ability to replace tobacco cigarettes, governmental regulation, its harm, its reliance potential, and whether the participant would refer to an e-cigarette user as a smoker.

The knowledge section consisted of 15 true or false questions. It was created based on a review of the literature and consultation with experts in the field of smoking and quitting smoking. Information regarding e-cigarettes, their efficacy, risks, and benefits was compiled from various sources to create the knowledge section [3,11,15]. Each correct response in the knowledge section was worth one point. A score of 15 was the maximum possible score; the higher the score, the more knowledgeable the participant.

Evaluation of the questionnaire instrument

Expert clinicians reviewed and validated the questionnaire instrument and evaluated its lucidity and readability, including its face validity and whether any questions were difficult to comprehend. In addition, before administering the questionnaire on a larger scale, a pilot study with a small number of participants (10 individuals) was conducted to assess its clarity; the results confirmed that it was straightforward. This evaluation aimed to determine the clarity, comprehensibility, and suitability of the questions, as well as to ensure the appropriateness of the content and identify any possible misunderstandings. The questionnaire items underwent an assessment of content validity to guarantee that they adequately covered the relevant topic for the study. The accomplishment of this task involved engaging in a discussion with both a pulmonologist and a family physician to ensure that the questions adequately encompassed the relevant subject matter. The evaluation of face validity was performed to ascertain the accuracy of the questions in capturing the targeted constructs. Expert feedback was sought to guarantee the relevance and appropriateness of the questions. The internal consistency for the attitude scale was examined using Cronbach’s alpha measure (r = 0.786), which reflected the acceptable reliability level. We investigated the convergent and discriminant validity of the attitude and knowledge measures. The adequacy of the sample for factor analysis was assessed through the utilization of the Kaiser-Meyer-Olkin (KMO) measure of sampling adequacy and Bartlett’s test of sphericity. These statistical tests were employed to determine if the observed variables in the dataset exhibited significant correlations with one another. The KMO measure had a value of 0.853, indicating that the dataset was appropriate for conducting factor analysis. The obtained p-value for Bartlett’s test was less than 0.001, suggesting a statistically significant correlation in the data.

The attitude and knowledge sub-scale of the original questionnaire by Aghar et al. were previously validated [14]. The preliminary assessment and review of the attitude scale involved three specialists in smoking cessation. The scale was subsequently adjusted and finalized, taking into account their comments. This iterative process aimed to ensure that the scale was broad and detailed enough to effectively capture the participants’ views toward ECs from multiple viewpoints [14]. A validation study was conducted to assess the efficacy of the knowledge scale in differentiating between good and poor knowledge. The sample size consisted of 45 people, with 17 classified as experts in e-cigarettes and 28 categorized as non-experts. The 45 individuals under consideration in this context were distinct from the participants who were first recruited to validate the translation. The individuals designated as experts in this study were healthcare professionals specializing in family medicine and pulmonology, specifically trained and certified in the field of smoking cessation. Non-expert persons in Beirut were selected as participants using random street sampling. These individuals were specifically chosen based on their awareness of e-cigarettes, but their lack of knowledge regarding the subject matter was confirmed. The questionnaire was completed by both specialists and non-experts in the presence of one of the investigators [14].

Questionnaire translation

To confirm the accuracy of the questionnaire’s translation from English to Arabic, validation research was conducted [14]. In this study, a total of 26 individuals were approached and requested to complete the questionnaire in both English and Arabic languages. The findings revealed a strong internal consistency in the translation, as evidenced by a high overall average percent agreement of 95%. To validate the translation of the knowledge scale, the internal consistency between the English and Arabic versions of the scale was also evaluated [14]. The findings demonstrated a substantial and statistically significant level of agreement across all questions. The agreement was determined using Cohen’s kappa statistic, percent agreement, and assessing the statistical significance of the agreement. The question pertaining to the presence of nicotine in the majority of e-cigarettes exhibited the lowest level of agreement. The percentage of agreement for this question was approximately 85%, the kappa statistic was 0.72, and the p-value was less than 0.0001 [14]. Nevertheless, it is important to note that this kappa value was deemed to be significantly elevated according to Cohen’s classification of agreement levels. All remaining questions on the knowledge scale demonstrated a notable degree of internal consistency, with certain questions achieving a unanimous agreement rate of 100%. The findings indicate that the translation, conducted by a certified translator, was executed with precision, confirming the trustworthiness of the translated content [14].

Sample size

The following equation was used to estimate the sample size: (1.96^2×0.5×(1−0.5)(0.05)^2). Using a 95% confidence interval (Z-Score (Z) = 1.96), a standard deviation (SD) of 0.5, and an error margin of 5%, the minimum required sample size was 385 individuals.

Ethical approval

This study was approved by the Bioethics Committee of Scientific and Medical Research at Jeddah University, Jeddah, Saudi Arabia (reference number: UJ-REC-141).

Statistical analysis

Using descriptive statistics, the demographic characteristics of the participating individuals were described. The normality of the continuous variable was examined using histogram and normality measures (skewness and kurtosis). On this basis, continuous variables, such as the knowledge score of the participants, were depicted using the median and interquartile range as the data was non-normally distributed. We used percentages (frequencies) to report categorical data. Using the Mann-Whitney U test and the Kruskal-Wallis test, the median knowledge and attitude scores of various demographic groups were compared. Binary logistic regression analysis was used to identify predictors of being knowledgeable of and having a positive attitude toward e-cigarettes. The cut-off points used to identify the dummy variable for knowledge and positive attitude regression models were 75% (knowledge score of 11.3) and 69.2% (attitude score of 9), as recommended by the original study [14]. Multiple logistic regression analysis was used to address potential biases that might have resulted from the utilization of the convenience sampling technique. The multiple logistic regression was adjusted for age, gender, and monthly income level. Statistical significance was defined as a two-sided p-value of less than or equal to 0.05. SPSS version 27 was used to conduct the statistical analysis (IBM Corp., Armonk, NY, USA).

## Results

A total of 422 participants were involved in this study. More than half of the participants (69.0%) were females. Almost one-third of the study participants (33.4%) were aged 24-30 years. More than half of the participants (57.6%) were single. Almost half of the study participants (50.5%) reported that they held a bachelor’s degree. Around 29.4% of the participants reported that their monthly income category was more than 7,500 SAR. Around one-third of the study participants (34.6%) were unemployed and current smokers (37.2%). Only 10.0% of the participants reported having comorbidities (Table 1).

**Table 1:** Participants’ demographic characteristics. SAR: Saudi Arabia riyal

Variable	Frequency	Percentage
Gender		
Females	291	69.0%
Age groups		
18–23 years	133	31.5%
24–30 years	141	33.4%
31–40 years	92	21.8%
41–50 years	33	7.8%
51–60 years	17	4.0%
61 years and older	6	1.4%
Marital status		
Single	243	57.6%
Married	145	34.4%
Divorced	29	6.9%
Widowed	5	1.2%
Education level		
Secondary school level or lower	162	38.4%
Bachelor’s degree level	213	50.5%
Higher education level	47	11.1%
Monthly income level		
Less than 2,500 SAR	121	28.7%
2,500–5,000 SAR	105	24.9%
5,001–7,500 SAR	72	17.1%
More than 7500 SAR	124	29.4%
Employment status		
Retired	15	3.6%
Unemployed	146	34.6%
Work in the healthcare sector	41	9.7%
University student	89	21.1%
Work outside the healthcare sector	131	31.0%
Current smoker (yes)	157	37.2%
Have comorbidities (yes)	42	10.0%

Knowledge and attitude toward e-cigarettes

Table 2 presents the median knowledge and attitude toward e-cigarette scores stratified by participants’ demographic characteristics. The median knowledge score for the study participants was 13.00 (11.00-14.00), which reflects a high level of e-cigarette knowledge. The median knowledge score for the study participants ranged between 3.00 and 15.00. The median attitude score for the study participants was 3.00 (1.00-5.00), which reflects a negative attitude toward the use of e-cigarettes. The median attitude score for the study participants ranged between 0.00 and 12.00. There was no statistically significant difference in the median knowledge score based on the demographic characteristics of the study participants (p > 0.05). However, there was a statistically significant difference in the median attitude score based on participants’ age, marital status, and smoking status (p < 0.05).

**Table 2:** Median knowledge and attitude toward the e-cigarette score. SAR: Saudi Arabia riyal; *: p < 0.05; **: p < 0.01; ***: p < 0.001

Variable	Median knowledge score (interquartile range)	P-value	Median attitude score (interquartile range)	P-value
Gender				
Females	13.00 (3.00)	0.360	3.00 (4.00)	0.820
Males	13.00 (3.00)		2.00 (4.00)	
Age groups				
18–23 years	13.00 (3.00)	0.122	3.00 (4.00)	0.047*
24–30 years	13.00 (4.00)		3.00 (5.00)	
31–40 years	13.00 (3.00)		1.00 (4.50)	
41–50 years	12.00 (2.00)		1.00 (4.50)	
51–60 years	13.00 (2.00)		2.00 (3.50)	
61 years and older	12.00 (7.50)		4.00 (6.00)	
Marital status				
Single	13.00 (3.00)	0.815	3.00 (5.00)	0.004**
Married	13.00 (3.00)		2.00 (4.00)	
Divorced	13.00 (3.00)		2.00 (4.50)	
Widowed	9.00 (8.00)		5.00 (3.50)	
Education level				
Secondary school level or lower	13.00 (4.00)	0.572	3.00 (4.00)	0.551
Bachelor’s degree level	13.00 (3.00)		2.00 (4.50)	
Higher education level	13.00 (3.00)		3.00 (6.00)	
Monthly income level				
Less than 2,500 SAR	12.00 (4.00)	0.533	3.00 (5.00)	0.249
2,500–5,000 SAR	13.00 (3.00)		3.00 (4.00)	
5,001–7,500 SAR	13.00 (2.75)		2.00 (4.00)	
More than 7,500 SAR	13.00 (3.00)		2.00 (6.00)	
Employment status				
Retired	12.00 (7.00)	0.413	3.00 (3.00)	0.178
Unemployed	12.00 (3.00)		3.00 (4.00)	
Work in the healthcare sector	13.00 (4.00)		1.00 (5.50)	
University student	13.00 (2.00)		2.00 (4.00)	
Work outside the healthcare sector	13.00 (3.00)		3.00 (5.00)	
Current smoker				
No	13.00 (3.00)	0.314	2.00 (4.00)	<0.001***
Yes	12.00 (4.00)		4.00 (6.00)	
Have comorbidities				
No	13.00 (3.00)	0.361	3.00 (4.00)	0.917
Yes	12.00 (3.00)		3.00 (6.00)	

Predictors of better knowledge and positive attitude

Table 3 presents the findings of the binary logistic regression analysis. Participants with a monthly income of 5,001 SAR and above were more likely to be knowledgeable about e-cigarette use (p < 0.05). However, binary logistic regression analysis did not identify any statistically significant predictor of positive attitude toward the use of e-cigarettes (p > 0.05).

**Table 3:** Predictors of better knowledge and positive attitude. SAR: Saudi Arabia riyal; *: p < 0.05; p-value was estimated using binary logistic regression analysis. Wald test was performed to demonstrate the global p-value for the variables.

Variable	Odds of being knowledgeable (95% confidence interval)	P-value	Odds of having a positive attitude score (95% confidence interval)	P-value
Gender				
Females (reference category)	1.00		1.00	
Males	1.40 (0.89–2.21)	0.143	0.79 (0.28–2.23)	0.649
Age groups: Knowledge section: (Wald test: 0.161; p-value: 0.688); attitude section: (Wald test: 2.514; p-value: 0.113)				
18–23 years (reference category)	1.00		1.00	
24–30 years	1.06 (0.64–1.74)	0.827	1.54 (0.49–4.83)	0.459
31–40 years	1.15 (0.65–2.02)	0.632	1.47 (0.41–5.24)	0.551
41–50 years	1.22 (0.53–2.77)	0.642	-	-
51–60 years	2.47 (0.67–9.03)	0.172	-	-
61 years and older	1.06 (0.19–5.99)	0.950	5.12 (0.50–52.39)	0.169
Marital status: Knowledge section: (Wald test: 1.249; p-value: 0.264); attitude section: (Wald test: 2.514; p-value: 0.113)				
Single (reference category)	1.00		1.00	
Married	1.06 (0.68–1.64)	0.807	1.36 (0.52–3.53)	0.527
Divorced	1.29 (0.55–3.04)	0.562	0.83 (0.10–6.75)	0.863
Widowed	0.33 (0.05–2.00)	0.226	-	-
Education level: Knowledge section: (Wald test: 0.900; p-value: 0.343); attitude section: (Wald test: 1.234; p-value: 0.267)				
Secondary school level or lower (reference category)	1.00		1.00	
Bachelor’s degree level	1.18 (0.77–1.82)	0.447	1.55 (0.52–4.62)	0.434
Higher education level	1.42 (0.69–2.91)	0.337	2.92 (0.75–11.35)	0.122
Monthly income level: Knowledge section: (Wald test: 7.684; p-value: 0.006); attitude section: (Wald test: 3.356; p-value: 0.067)				
Less than 2500 SAR (reference category)	1.00		1.00	
2,500–5,000 SAR	1.59 (0.92–2.75)	0.097	0.65 (0.18–2.27)	0.494
5,001–7,500 SAR	2.19 (1.15–4.16)	0.017*	0.71 (0.18–2.83)	0.625
More than 7,500 SAR	1.85 (1.09–3.15)	0.023*	0.68 (0.21–2.22)	0.527
Employment status: Knowledge section: (Wald test: 1.877; p-value: 0.171); attitude section: (Wald test: 0.275; p-value: 0.600)				
Retired (Reference category)	1.00		1.00	
Unemployed	1.07 (0.36–3.17)	0.901	-	-
Work in the healthcare sector	1.29 (0.38–4.35)	0.686	-	-
University student	2.30 (0.73–7.24)	0.155	-	-
Work outside the healthcare sector	1.46 (0.49–4.38)	0.496	-	-
Current smoker				
No (reference category)	1.00		1.00	
Yes	0.69 (0.46–1.05)	0.085	1.55 (0.62–3.90)	0.352
Have comorbidities				
No (reference category)	1.00		1.00	
Yes	1.08 (0.54–2.15)	0.825	1.07 (0.24–4.79)	0.932

Table 4 presents the findings of the multiple logistic regression analysis. The regression model was adjusted for age, gender, and monthly income level. Participants working outside the healthcare sector were 56.0% less likely to be knowledgeable about e-cigarette use and 2.3 folds more likely to have a positive attitude toward using it (p < 0.05). Besides, current smokers were 43.0% less likely to be knowledgeable about e-cigarette use and 2.9 folds more likely to have a positive attitude toward using it (p < 0.05).

**Table 4:** Multiple logistic regression analysis. Multiple logistic regression analysis adjusted for age, gender, and monthly income level; *: p < 0.05; ***: p < 0.001

Variable	Odds of being knowledgeable (95% confidence interval)	P-value	Odds of having a positive attitude score (95% confidence interval)	P-value
Marital status				
Single (reference category)	1.00		1.00	
Married	0.74 (0.11–5.01)	0.758	-	
Divorced	0.77 (0.11–5.24)	0.787	-	
Widowed	0.40 (0.05–3.18)	0.389	-	
Education level				
Secondary school level or lower (reference category)	1.00		1.00	
Bachelor’s degree level	0.96 (0.46–1.99)	0.913	1.25 (0.60–2.57)	0.552
Higher education level	0.98 (0.50–1.91)	0.948	1.73 (0.88–3.38)	0.111
Employment status				
Retired (reference category)	1.00		1.00	
Unemployed	2.23 (0.52–9.52)	0.280	0.86 (0.22–3.40)	0.827
Work in the healthcare sector	0.79 (0.46–1.36)	0.395	1.27 (0.74–2.16)	0.390
University student	1.31 (0.63–2.72)	0.469	2.12 (1.00–4.53)	0.051
Work outside the healthcare sector	0.44 (0.23–0.85)	0.015*	2.25 (1.19–4.27)	0.013*
Current smoker				
No (Reference category)	1.00		1.00	
Yes	0.57 (0.38–0.88)	0.011*	2.89 (1.85–4.51)	<0.001***
Have comorbidities				
No (reference category)	1.00		1.00	
Yes	0.63 (0.31–1.27)	0.195	1.33 (0.66–2.67)	0.422

## Discussion

This study aimed to investigate and understand the knowledge and attitude regarding the use of e-cigarettes in Saudi Arabia. The study participants exhibited a substantial level of e-cigarette knowledge, with a median knowledge score of 13.00 (11.00 to 14.00). Similarly, a considerable level of knowledge was found in Egypt [16], where 41% of study participants believed that e-cigarettes aid smoking cessation and about 31.9% considered it less harmful than traditional cigarettes. Meanwhile, in Saudi Arabia, e-cigarette awareness was higher among the study participants, and its use was popular among non-smokers, potentially leading to an increase in smoking prevalence [17]. Indeed, it has been found that regulating e-cigarette advertisements in the media is necessary because our understanding of the effectiveness and possible risks of these products is still limited in the scientific community [18].

In our study, the high level of knowledge demonstrated by the participants was accompanied by a negative attitude toward the use of e-cigarettes. Similar results were reported by a previous study in Australia [19], indicating fair health literacy regarding the use of e-cigarettes. However, there is an intriguing question about the factors influencing their attitudes. Indeed, potential factors could include health concerns and social perceptions of e-cigarette use. In a study from the United Kingdom, 63% of the participants agreed that e-cigarettes are bad for health and 75% agreed that it is addictive. Moreover, one out of ten participants reported that they were currently using e-cigarettes on a daily basis [20]. Indeed, the social stigma around e-cigarette use could make it harder for people to quit smoking. It has been reported that e-cigarette users often seek recognition for trying to reduce harm, and if they do not get acknowledgment for their efforts, they might go back to smoking cigarettes [21]. Therefore, further qualitative research might be necessary to delve deeper into these attitudes, exploring the underlying reasons that shape participants’ negative perceptions.

People’s views on e-cigarettes might differ based on their age, whether they are married or single, and if they smoke. This means younger people, married individuals, and smokers have different attitudes towards e-cigarettes, likely due to their personal experiences and backgrounds. It has been reported that among teenagers, e-cigarette use is common because these devices are easy to get, seen as healthier than regular cigarettes, and are attractive in appearance [22]. Further, other factors such as curiosity and having friends using e-cigarettes were found to affect the attitude toward the initiation of e-cigarette use [20]. However, in adult smokers with substance use disorders, those who used both e-cigarettes and regular cigarettes were more likely to have attempted quitting smoking in the past year. They also preferred using e-cigarettes over nicotine patches or gum for their attempts at quitting [23].

In this study, we found that having a higher monthly income (5,001 SAR or more) was linked to greater knowledge about the use of e-cigarettes. Interestingly, it was found that those with higher monthly income were associated with a higher prevalence of e-cigarette usage [24]. However, it has been suggested that individuals with higher socioeconomic status might have better access to educational resources or are more receptive to health-related information [25]. Moreover, this raises concerns about potential disparities in health knowledge among different socioeconomic groups, highlighting the importance of targeted health education campaigns to bridge this gap.

Based on the findings of this study, targeted public health campaigns are crucial to increasing awareness about e-cigarette use in Saudi Arabia, especially among young people and non-smokers. These campaigns should focus on the health risks associated with e-cigarettes and counter the perception that they are a safe alternative to traditional cigarettes. Additionally, there is a need for in-depth qualitative research to understand the factors influencing negative attitudes, especially among specific demographic groups. Regulation of e-cigarette advertisements, particularly those targeting youth, is essential to prevent glamorization. Further, efforts to address socioeconomic disparities in e-cigarette knowledge and usage through accessible education are necessary. Healthcare providers should also be educated about the potential role of e-cigarettes in smoking cessation to provide tailored support to smokers, where implementing these measures can help reduce e-cigarette usage and its impact on smoking rates in the country.

This study has a few limitations. The cross-sectional study design restricted our ability to examine causal relationships between the study variables. By capturing data at a single moment, cross-sectional studies are limited in their ability to analyze trends or changes in variables across time. The use of an online survey study design might have limited the generalizability of our study findings as some of the intended study samples might not have had access to the internet or social media websites (which is known as selection bias). However, based on the latest available statistical data from 2023, it has been observed that around 79.3% of the general population in Saudi Arabia actively engages with social media platforms. Therefore, we assume that this would increase the generalizability of our study findings. Although convenience sampling is frequently chosen for its efficiency and affordability, it has the potential to introduce bias into the sample, compromising its ability to correctly represent the larger population. Non-response bias constitutes an additional constraint for this study, as online surveys frequently encounter non-response bias when particular persons or groups exhibit a lower likelihood of participation. Cross-sectional surveys mainly depend on self-reported data, making them susceptible to recall bias and social desirability bias. Therefore, our study findings should be interpreted carefully.

## Conclusions

Our study sample showed a high level of e-cigarette knowledge accompanied by a negative attitude toward the use of e-cigarettes. Individuals’ income level was an important predictor of e-cigarette knowledge. A comprehensive qualitative study approach is necessary to gain a thorough understanding of the various factors that contribute to unfavorable attitudes toward the use of e-cigarettes, particularly within certain demographic cohorts.
